# Self-administration of medication during hospitalization—a randomized pilot study

**DOI:** 10.1186/s40814-020-00665-3

**Published:** 2020-08-18

**Authors:** Charlotte Arp Sørensen, Charlotte Olesen, Marianne Lisby, Ulrika Enemark, Annette de Thurah

**Affiliations:** 1 Hospital Pharmacy Central Denmark Region, Aarhus, Denmark; 2 Medical Department, Randers Regional Hospital, Dronningborg Boulevard 16D, 8930 Randers, NØ Denmark; 3 Department of Clinical Medicine, Aarhus University, Aarhus, Denmark; 4 Clinical Pharmacy, Aarhus University Hospital, Aarhus, Denmark; 5 Research Centre for Emergency Medicine, Aarhus University Hospital, Aarhus, Denmark; 6 Department of Public Health, Aarhus University, Aarhus, Denmark; 7 Department of Rheumatology, Aarhus University Hospital, Aarhus, Denmark

**Keywords:** Medication error, Clinical safety, Patient involvement, Feasibility, Self-administration, Self-management

## Abstract

**Background:**

Self-administration of medication (SAM) during hospitalization is a complex intervention where patients are involved in their course of treatment. The study aim was to pilot test the SAM intervention. The objectives were to assess the feasibility of conducting a randomized controlled trial on the safety and cost-consequences of SAM during hospitalization.

**Methods:**

The study was performed in a Danish cardiology unit.

Patients ≥ 18 years capable of self-administering medication during hospitalization were eligible. Patients were excluded if they did not self-administer medication at home, were incapable of self-administering medication, were not prescribed medication suitable for self-administration, did not bring their medication, or were unable to speak Danish.

Feasibility was assessed as part of the pilot study. A future randomized controlled trial was considered feasible if it was possible to recruit 60 patients within 3 months, if outcome measurement method was capable of detecting dispensing errors in both groups, and if patients in the intervention group were more satisfied with the medication management during hospitalization compared to the control group.

Forty patients were recruited to gain experience about the intervention (self-administration). Additionally, 20 patients were randomized to the intervention or control group (nurse-led dispensing) to gain experience about the randomization procedure.

Dispensing error proportions were based on data collected through disguised observation of patients and nurses during dispensing. The error proportion in the control group was used for the sample size calculation. Patient acceptability was assessed through telephone calls.

**Results:**

Of the 60 patients recruited, one withdrew and 11 were discharged before observation resulting in analysis of 39 patients in the intervention group and nine in the control group. A dispensing error proportion of 3.4% was found in the intervention group and 16.1% in the control group. A total of 91.7% of patients in the intervention group and 66.7% in the control group were highly satisfied with the medication management during hospitalization. The overall protocol worked as planned. Minor changes in exclusion criteria, intervention, and outcome measures were considered.

**Conclusions:**

It may be feasible to perform a pragmatic randomized controlled trial of the safety and cost-consequences of self-administration of medication during hospitalization.

**Trial registration:**

ClinicalTrials.gov, NCT03541421, retrospectively registered on 30 May 2018.

## Background

Medication administration errors (MAEs) in healthcare settings occur in approximately 20% of the total opportunities for error (OEs) [1, 2], and like other medication errors, some of them may cause patient discomfort and harm and thereby increase length of hospital stay, healthcare costs, and mortality [3]. MAEs are defined as the administration of a dose of medication that deviates from the prescription, from hospital guidelines, or from written procedures [1, 4, 5], and the term MAEs often covers errors both in the process of nurse-led dispensing in the medicine room and administration to the patient (with, for example, patient identification) [1, 5]. It has been suggested that some of the errors may be avoided through patient involvement, where patients and health professionals work in partnership [6, 7]. Involving patients as active partners may improve their knowledge, skills, and confidence in self-managing their health condition [7].

The use of medication is an aspect of self-management [8, 9], and self-administration of medication (SAM) during hospitalization may give the patients the opportunity to continue their medication management routines from home [10]. SAM in hospital means that selected patients are responsible for storing and administering their own medication with the nurse acting as supervisor of the process [11]. SAM is associated with a number of advantages including independence, cooperation, increased knowledge, and empowerment [12–14]. A recent Danish study reports effects on clinical outcomes as pain scale score and the consumption of analgesics as well [15]. Disadvantages may be overdose, underdose, and non-adherence [12–14]. Previous studies have investigated safety as adherence or medication errors caused by patients. Different outcome measurement methods have been used, including pill count, patients’ self-reported adherence, urine sampling, and disguised observation [13]. Studies have shown conflicting results when SAM is compared with medication dispensing performed by a nurse/technician in the medicine room [13]. Hence, the evidence on safety in SAM is unclear. Many of the studies have methodological flaws such as variable definitions, small sample sizes, and inadequate reporting of results. Therefore, a well-designed study in larger scale is needed [12–14]. A SAM intervention will systematically remove the majority of the administration errors by nurses (e.g., patient identification, wrong patient). A Danish study found patient identification errors to comprise 88% of the observed errors during the administration of medication [5]. Hence, the primary focus of the intervention was to explore the difference in safety when comparing nurse- and patient-led medication processes. Therefore, we chose to measure the proportion of errors during medication dispensing. We considered that the responsibility for the medication management belongs to the patients when introducing SAM. However, patients, nurses, and doctors are involved in different steps: assessment of the patient’s capability of self-administration, assessment of the patient’s personal medication supplies [16], and providing medication as supplement to medication brought to hospital. It involves support, education, and communication between the patient and the doctor/nurse when prescription changes are made. Therefore, research and evaluation within SAM must be considered a complex intervention [17]. In complex interventions, a feasibility and pilot study is recommended to address methodological, procedural, and clinical uncertainties of the intervention and the study design before conducting a randomized controlled trial (RCT) [18].

The study aim was to pilot test the SAM intervention. The objectives were to assess the feasibility of conducting an RCT on the safety and cost-consequences of SAM during hospitalization in terms of recruitment, chosen outcome measurement methods, and patient acceptability.

## Methods

This study is reported following the checklist from the CONSORT 2010 statement: extension to randomized pilot and feasibility trials [19]. See Additional file 1.

### Setting

The study was performed at the Medical Department (Cardiology Unit, 28 beds), Randers Regional Hospital, Denmark, within a catchment area of 225,000 inhabitants. The unit has 2000 admissions per year and provides basic cardiology service.

Medication reconciliation and updating the electronic Medication Administration Record (eMAR) are performed by doctors at, for example, the Emergency Department, Randers Regional Hospital, prior to the referral to the Cardiology Unit.

The population in Denmark enjoys access to free tax-supported healthcare including outpatient admission at hospitals [20]. It is permitted to ask patients to use their medication during hospitalization.

Doctors are responsible for prescriptions and nurses for dispensing and administration of medication from the medicine room. Pharmaconomists (pharmacy technician with a 3-year degree (180 European Credit Transfer System points) [21]) are responsible for the medicine room supply, but pharmacists are not at the wards on daily basis.

### Sampling

Patients were consecutively recruited Monday to Thursday by the primary investigator (PI) (CAS) or a nurse. Written informed consent was obtained prior to participation. Patients were eligible for inclusion if they were ≥ 18 years, were prescribed at least one medication suitable for self-administration, and were capable of self-administering medication at hospital.

Patients were excluded if they did not self-administer medication at home, were incapable of self-administering medication at hospital, were not prescribed medication suitable for self-administration, did not bring any of their medication to hospital, or were unable to speak Danish.

Assessment of capability of self-administration was delegated from doctors to nurses. The assessment was based on an evaluation of the patient’s current situation including the cognitive, emotional, and health status [22]. A number of questions were asked during the assessment: (i) Do the patient dispense and self-administer own medication at home? (ii) Is the patient capable of self-administration of medication during hospitalization (professional assessment, e.g., oriented in time and place, not confused; able to read and understand text on labels and papers)? (iii) Do the patient know when, how, and why usual medication should be taken (professional assessment)? (iv) Is the patient able to open medication containers and blister cards? (v) Is there any other risk factor that excludes the patient from self-administration (e.g., a history of alcohol/drug abuse)?

A target of 60 patients was considered appropriate to give meaningful results of the processes [17]. The initial 40 patients were recruited to gain experience about the intervention (self-administration). The last 20 patients were randomized to the intervention or control group (nurse-led medication dispensing) at a 1:1 ratio to gain experience about the randomization procedure. Randomization was performed by the Hospital Pharmacy’s Department of Quality Assurance on the webpage randomization.com. For each participant, they wrote the allocation down and placed it in a sealed opaque envelope. Due to the type of intervention, blinding was not possible in this study.

### Intervention

#### Development and training

A project group comprising nurses (Cardiology Unit), a senior doctor (Cardiology Unit), a pharmaconomist (Hospital Pharmacy), a pharmacist (Hospital Pharmacy), and the PI was established to develop the intervention. The project group worked in subgroups under the headings: Patient, Medication, Storage, Documentation, and Communication. The intervention was developed from November 2016 to March 2017 based on a literature search performed by the PI as well as on information on Danish experiences with SAM from Hvidovre Hospital [22], Regional Hospital West Jutland (unpublished), and Regional Hospital Central Jutland (unpublished). The PI provided structured training on the intervention to participating nurses from the Cardiology Unit in March 2017. Medical doctors were informed about the intervention as well.

#### Description of the intervention

**Fig. 1:**
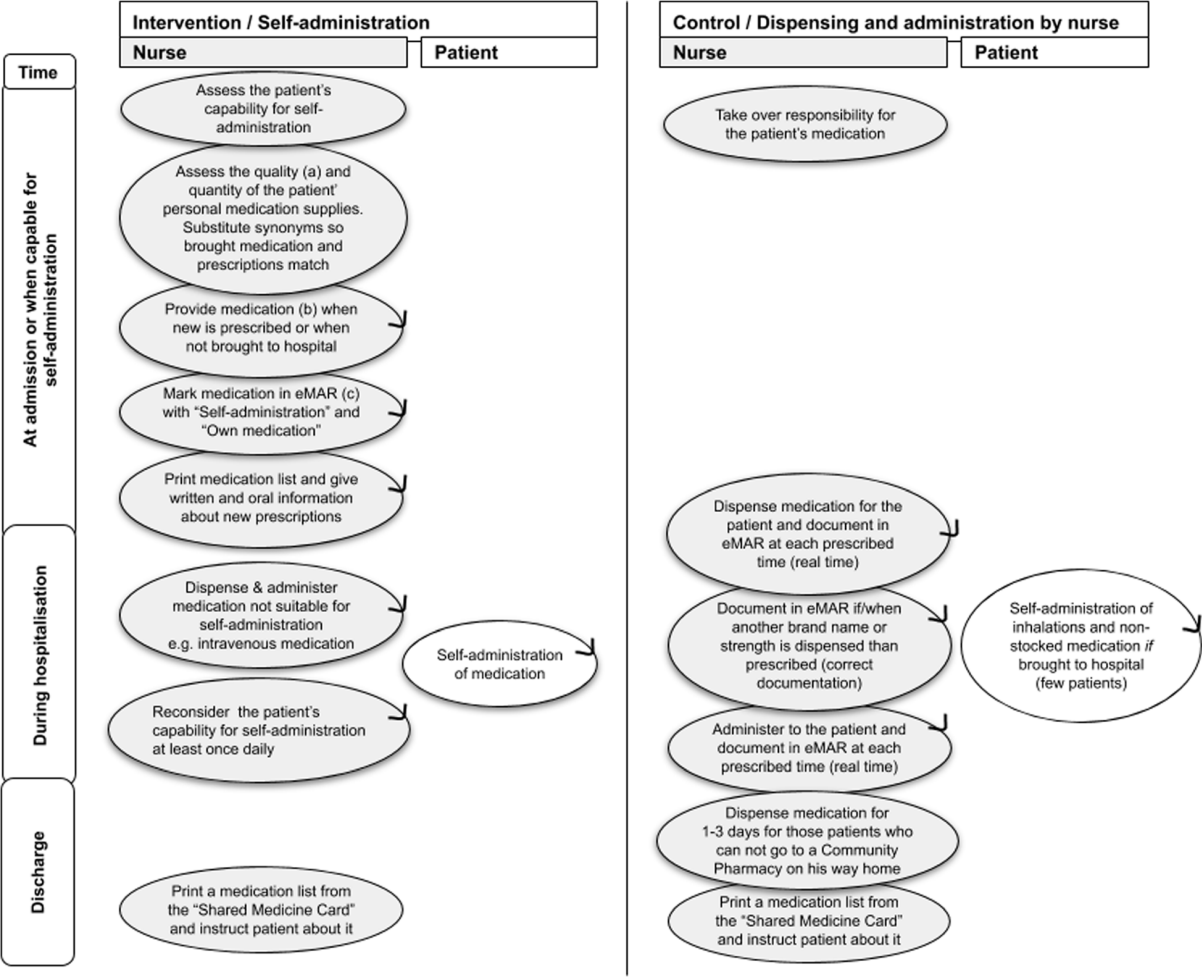
Workflows. (a) By assessing the appearance, container, labeling, identification of content, storage conditions, and expiration [16]. (b) Medication to the patient was delivered from the medicine room as the smallest/cheapest original package. (c) eMAR, electronic Medication Administration Record. An arrow on the circle indicates that the process can be repeated depending on medication changes and the patient’s length of hospital stay

The patient’s personal medication supplies were assessed by a nurse in relation to quality (e.g., expiry date) and quantity (number of tablets) [16] and were compared to prescriptions in the eMAR. Any uncertainty was conferred with a doctor. Medication was provided by the hospital and delivered from the medicine room as a whole package labeled with the patient’s name and Civil Registration Number if new medication was prescribed or if the patient had not brought the medication to hospital. A nurse was responsible for delivering the right medication and for the other nursing tasks of the intervention (Fig. 1).

Inspired by “The green bag scheme” from England [23], medication in use during hospitalization was placed in a green bag. Any brought medication not in use was placed in a red bag. Medication was stored in the patient wardrobe, and the key was kept by the patient. The patient was responsible for the self-administration of medication during hospitalization except for medication not suitable for self-administration. These were “once only prescriptions,” medication stored in the refrigerator (except insulin), inhalations through nebulization, injections and infusions, and variable high dose of digoxin during digitalization. All other administration forms and prescriptions were considered suitable for self-administration, including prescriptions of medication “as needed.”

### Control group

Medication was dispensed by a nurse in the medicine room (Fig. 1). Patients were allowed to self-administer inhalation and non-stocked medication brought to the hospital; however, most medication was provided by the hospital (91% (52/57 prescribed medication)).

### Outcome assessment

#### Feasibility

The study was a 3-month follow-up study where feasibility was assessed as part of the pilot study. The list of methodological issues for feasibility and pilot studies recommended by Shanyinde et al. [24] and Bugge et al. [25] was used to categorize and assess relevant design issues. Two of 14 issues were not relevant for this study (blinding and logistics of a multi-center trial) and were thus excluded. Due to the type of intervention and patient safety, blinding was not a possibility.

A future RCT was considered feasible if it was possible to recruit 60 patients within 3 months, if the outcome measurement method was capable of detecting dispensing errors in both groups, and if patients in the intervention group were more satisfied with the medication management during hospitalization compared to the control group.

#### Primary outcome

The primary outcome was the proportion of dispensing errors in relation to the total number of opportunities for error (OEs) observed. Dispensing errors were defined as the dispensing of a dose of medication that deviates from the prescription, from hospital guidelines, or from written procedures. This definition was derived from the definition of MAEs [1, 4, 5]. An OE was defined as any dose dispensed plus any dose prescribed but omitted [4, 5]. The number of observed OEs was registered after each observation and summed to a total number for the group and a mean number per patient.

The dispensing error proportion was calculated using the formula: $\frac{\mathrm{Dispensing}\kern0.5em \mathrm{errors}}{\mathrm{OEs}}\times 100\%$.

Errors were divided into clinical and procedural errors as described in the work of Westbrook et al. [26] and Risør et al. [5]. A clinical error occurred when the patient did not receive the medication as prescribed in the eMAR [5]. A procedural error occurred when the nurse deviated from written procedures or guidelines [5]. Deviations could potentially lead to medication errors [5, 26]. Common types of errors derived from the literature are described in Table 1 [4, 5, 27].

**Table 1:** Error types

Error type	Definition
**Clinical errors**	
Wrong medication	The dispensed medication was not prescribed in the eMAR.
Omission of dose	The prescribed dose of the medication was not dispensed to the patient.
Wrong dose	The dose deviated from the prescribed dose.
Wrong administration form	The form of the dispensed medication deviated from the eMAR prescription.
**Procedural errors**	
Wrong strength per unit	The strength of the dispensed medication deviated from the prescription in the eMAR. For example, 1 tablet of 100 mg was prescribed in the eMAR but 2 tablets of 50 mg were dispensed. If this deviation was not documented in the eMAR, it was regarded a procedural error.
Lack of documentation of a substitution	A substitution was made but not documented in the eMAR.
Lack of documentation of the dispensing	The medication was not documented as “dispensed” in the eMAR.

Sources: relevant error types derived from the literature [4, 5, 27]

Dispensing of medication from original containers into medicine cups/dosage boxes was observed by the PI using a modified disguised observation technique [4, 28–30]. Observations of nurses and timekeeping were performed in the medicine room each morning. The nurses were aware of timekeeping, but were unaware of the observation’s real purpose. Observations of patients were performed in the patient room when they filled a medicine cup with their morning medicine or a dosage box with medication for the next 24 h after the ward round (only medication prescribed for intake during the day shift was noted as observations). The patients were unaware of the purpose of the observation. Observations were performed Monday to Friday and were repeated for as many days as possible to check for errors caused by prescription changes. Observations were registered, entered into Excel (Microsoft 2010), and compared with prescriptions in the eMAR and written procedures. Deviations were recorded and errors were categorized (Table 1). The observer only intervened if a severe error was observed.

#### Secondary outcomes

Telephone calls were performed by the PI 2 weeks after discharge to explore patient acceptability and the number of readmissions and contacts to general practitioners (GPs) in both groups. A structured guide of questions was used (Additional file 2).

Cost calculations and time measurements were performed to gain knowledge of data needs and collection methods for the cost-consequence analysis (hospital perspective). The processes within the intervention were described in Fig. 1; from these processes, it was found that costs were related to the use of medication, materials, and nursing time spent on different tasks. Costs for providing medication and materials (medicine cups, plastic bags, dosage boxes) were calculated on micro-costing level for each patient based on information from the eMAR and observations. Medication cost and material cost per patient were calculated by multiplying quantity and unit cost (based on respectively hospital pharmacy prices and Central Denmark Region prices). Nurse time spent on dispensing and administration in the control group and SAM start-up (e.g., assessment of the patient’s personal medication supplies, documentation in the eMAR, and instructions to the patient) in the intervention group was measured with stopwatches.

### Analysis methods

Data were analyzed using descriptive statistics.

Student’s *t* test or the Wilcoxon rank-sum test was used to compare continuous outcomes.

Length of stay in the hospital was registered as a baseline characteristic. The distribution of length of stay is skewed positively, so data were log-transformed prior to analysis.

Chi-squared test or Fisher’s exact test was used to compare binary outcomes.

Sample size for the RCT was calculated in Stata15 (StataCorp, 4905 Lakeway Drive, TX, USA) based on the observed dispensing error proportion in the control group.

## Results

Assessment of the 12 methodological issues is summarized in Table 2 and described in more details in the text. The methodological issues are presented in accordance with the work of Shanyinde et al. [24] and Bugge et al. [25]. Subheading numbers refer to the item number in Table 2.

**Table 2:** Summary—assessment of the 12 methodological issues for feasibility studies

Methodological issues	Findings	Evidence
1) Did the feasibility/pilot study allow a sample size calculation for the main trial?	Achieved from the error rate in the control group.	Error proportion 16.1% Sample size: observation of 1020 OEs per group
2) What factors influenced eligibility and what proportion of those approached was eligible?	Ineligibility was mainly due to the following: patients not being capable of self-administration, not self-administering at home, and capable but not bringing own medications.	153/441 patients assessed were eligible, see Fig. 2.
3) Was recruitment successful?	Recruitment was satisfactory.	60 patients were recruited within 3 months as planned.
4) Did eligible participants consent?	Invited patients consented.	10/70 patients declined
5) Were participants successfully randomized and did randomization yield equality in groups?	Participants were successfully randomized with equality in groups.	No statistically significant differences between groups, e.g., gender (*p* = 1.0), age (*p* = 0.82), and length of stay (*p* = 0.51). See Table 3.
6) Did participants adhere to the intervention?	Good adherence, but not all procedures worked in clinical practice (participants = nurses).	Nurses delivered less medication to patients in the intervention group than planned. Nurses did not remove the “self-administration status” from the eMAR at discharge as planned
7) Was the intervention acceptable to the participants?	The intervention was well accepted by patients.	Highly satisfied—91.7% I; 66.7% C Prefer future SAM—94.4% I; 66.7% C
8) Was it possible to calculate intervention costs and duration?	Further details in the calculation of costs and time measurements must be taken into account. Recruitment period was estimated to be 14 months.	Time used on other tasks than medication dispensing, administration, and SAM start must be measured.
9) Were outcome assessments completed?	Main areas of interest were assessed.	Dispensing errors were observed in both groups with an error proportion of 3.4% in the intervention group and 16.1% in the control group. See Table 3.
10) Were outcomes measured those that were the most appropriate outcomes?	Primary outcome was appropriate. It would be relevant to add secondary outcomes of SAM’s effects after discharge, e.g., perceptions regarding medication.	The proportion of dispensing errors in relation to OEs in total is appropriate as primary outcome for the safety of SAM.
11) Was retention to the study good?	Retention was good.	Response rate follow-up—92.3 % I; 100 % C
12) Did all components of the protocol work together?	The overall protocol worked as planned. Few adjustments were considered.	Adjustments were considered for exclusion criteria, recruitment procedure, intervention, and outcome methods (see numbers 1, 6, and 8 in this table)

*I* intervention, *C* control

Sources: methodological issues based on and presented in the order of the work of Shanyinde et al. [24] and Bugge et al. [25]

### Outcome assessment and sample size calculation (Table 2, items 1 and 9)

The dispensing process was observed in *n* = 39 (78% (39/50)) patients in the intervention group and in *n* = 9 (90 % (9/10)) patients in the control group. There were no statistical differences in baseline characteristics between groups (Table 3), and thus, the randomization procedure of the 20 patients was considered successful.

**Table 3:** Baseline characteristics and observation of dispensing

	Intervention, *n* = 39	Control, ***n*** = 9
**Baseline characteristics**		
Proportion males, number (%)	26 (67)	6 (67)
Age, mean (SD)	64.8 (11.7)	65.8 (11.9)
Lives alone, number (%)	16 (41)	1(11)
Proportion “no medication prior to admission,” number (%)	5 (12)	1 (11)
Number of medication at admission, mean (SD)	5.6 (4.2)	2.6 (1.5)
Number of medication at discharge, mean (SD)	7.8 (3.4)	7.4 (2.3)
Length of stay, median (SD)	3.2 (2.0)	2.7 (1.4)
Cardiology patient, number (%)	31 (79)	7 (78)
**Observation of dispensing**		
**Observation numbers**		
Patients where dispensing was observed 1 time, number	39	9
Patients where dispensing was observed 2 times, number	25	2
Patients where dispensing was observed 3 times, number	14	2
Patients where dispensing was observed 4 times, number	8	1
**Opportunities for error**		
OEs, total number	348	56
OEs at observation 1, mean (range)	4.6 (1–9)	4.3 (1–9)
**Error proportion**		
Total, number (%)	12 (3.4)	9 (16.1)
Clinical errors, number (%)	12 (3.4)	0
Procedural errors, number (%)	Not checked	9 (16.1)
**Error type**		
Omission, number	5	0
Wrong dose, number	7	0
Lack of documentation of a substitution, number	Not checked	4
Wrong strength per unit, number	Not checked	4
Lack of documentation of a dispensing, number	0	1

*SD* standard deviation

Baseline characteristics: no statistically significant differences between groups were observed

During the first observation, a mean of 4.6 OEs per patient (95% CI 3.9; 5.4) was observed in the intervention group and 4.3 OEs per patient (95% CI 2.5; 6.2) in the control group.

Observation of the dispensing process was possible from one to four times according to the patients’ length of hospital stay.

A dispensing error proportion of 3.4% (12/348) was observed in the intervention group and of 16.1% (9/56) in the control group. Procedural errors were not measured in the intervention group as this initially was considered irrelevant (Table 3).

#### Sample size calculation

With an error rate of 16% and a reduction of 30% considered clinically relevant [5], an observation of 1020 OEs in each group will be required in the RCT.

### Eligibility (Table 2, item 2)

**Fig. 2:**
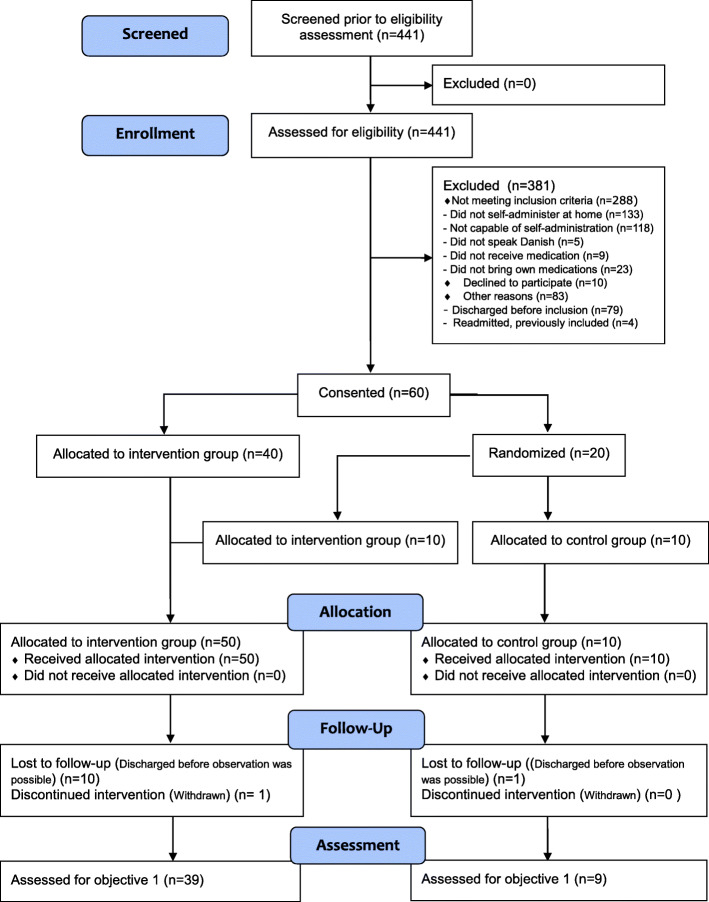
Flow diagram (CONSORT [19])

A total of 512 patients were admitted to the unit during the study period (March 6 to June 12, 2017, with 46 inclusion days); 34.7% ((60 + 10 + 83)/441) of the assessed patients were eligible (Fig. 2).

### Recruitment (Table 2, item 3)

A total of 60 patients were recruited within 3 months. One patient withdrew because of a change in his capability to self-administer medication (delirium). Eleven patients were discharged before observation of dispensing.

In total, 8.0% (23/288) of the excluded patients were capable of self-administration, but did not bring their medication to hospital.

Nurses sometimes forgot to assess new patients’ eligibility, so eligibility was assessed in the days after and often too late. Hence, 51.6% (79/(60 + 10 + 83)) of the eligible patients were discharged before recruitment was possible.

### Consent (Table 2, item 4)

In total, 39% of the eligible patients consented to participate (60/(60 + 10 + 83)). A total of 14.3% (10/70) of the invited patients declined to participate. The reasons were not registered.

### Randomization procedure (Table 2, item 5)

The use of sealed opaque envelopes with the randomization allocation worked as planned in the 20 patients. It was easy to use, and group allocation was unknown until the opening of the envelopes. In a future RCT, random block sizes must be used to avoid prediction of group allocation.

### Adherence to intervention (Table 2, item 6)

Nurses were responsible for the main part of the intervention and adhered to most of the intervention; however, due to busyness, nurses missed some of the study procedures. Nurses delivered blister packages instead of whole packages when providing medication to patients in the intervention group. Nurses did not remove the registration “Self-administration” and “Own medication” from the eMAR at discharge. Thus, repeated reminders about the intervention workflow were considered necessary.

### Acceptability of intervention (Table 2, item 7)

In the intervention group, 92.3% of the patients (36/39) were reached by telephone, out of which 91.7% (33/36) were highly satisfied with the medication management during hospitalization and 94.4% (34/36) preferred self-administration in a possible future hospitalization. All patients in the control group were reached by telephone. A total of 66.7% (6/9) were highly satisfied with the medication management during hospitalization (nurse-led dispensing); however, actually 66.7% (6/9) expressed a wish for self-administration in a possible future hospitalization instead.

### Costs and duration of intervention (Table 2, item 8)

In the intervention group, there was a mean cost of 16.8 € for providing medication during hospitalization (range 0.1–69.9€, median 12.4 €) (except intravenous antibiotics). In the control group, the mean cost was 12.3 € (range 0.4–39.8 €, median 8.7 €).

In the intervention group, 18% (7/39) had a readmission and 26% (10/39) had a GP-contact since discharge. In the control group, 11% (1/9) had a readmission and 22% (2/9) had a GP-contact since discharge. We realized that costs and contacts within 30 days were more relevant to stakeholders than a date within 14 days; hence, information must come from registries instead.

A mean of 19.9 min per patient (SD 6.7 min) was used to start SAM in the intervention group including assessment of the patient’s personal medication supplies, documentation in the eMAR, and instruction of the patient. In the control group, it took a mean of 0.57 min to dispense one medication in the medicine room (SD 0.3 min) and 0.94 min (SD 0.9 min) to administer the medication to the patient (including the walk from the medicine room and patient identification). To compare the time used to deliver the intervention with the time used for dispensing and administering medication in the control group, the total number of doses and administrations (medication rounds per day per patient) each day must be registered in the RCT.

In the intervention group, dispensing was observed twice in two thirds of the patients (25/39) and once in one third of the patients (14/39) (Table 3). With a total number of 1020 OEs to be observed per group and a mean of 4.5 (4.3 + 4.6/2) OEs per observation, it was estimated that approximately 150 patients were needed per group. With six patients recruited a week plus time covering days off work, the recruitment period was estimated to be 14 months.

### Selection of most appropriate outcomes (Table 2, item 10)

The dispensing error proportion was considered appropriate as it indicated whether the patient has received and understood the instructions from the nurses. As secondary outcomes, it is relevant to explore the effects of SAM after discharge, e.g., perceptions about medication [31], which is correlated to medication adherence [32].

### Retention (Table 2, item 11)

Retention was good since the response rate at follow-up was 92.3% (36/39) in the intervention group and 100% in the control group.

### All components of the protocol work together (Table 2, item 12)

Overall, the protocol worked as planned. Challenges were mainly related to organizational aspects of the intervention; they will be further elaborated in the “Discussion” section.

## Discussion

This study aimed to pilot test self-administration of medication as compared to nurse-led medication dispensing. As part of the pilot study, an assessment of the feasibility of conducting an RCT on the safety and cost-consequences of SAM during hospitalization was performed.

The findings showed that many patients were capable of self-administering medication during hospitalization. A total of 60 patients were recruited within 3 months as planned, and thus, recruitment for an RCT was considered possible. It was possible to detect dispensing errors in both groups through modified disguised observation. The intervention was well accepted by patients, and in general, nurses adhered to the study protocol.

To the best of our knowledge, no feasibility and pilot studies within safety in SAM have been published yet, which makes it hard to compare to others.

### Complexity and procedures

The study confirmed that introducing SAM is complex involving both patients and healthcare professionals with nurses accounting for the largest part of the intervention. A recent study from Denmark has shown that nurses save 12 min per hospitalization when comparing self-administration to medication dispensed and administered by nurses [22]. In our study, some nurses initially felt that the intervention was a burden, and it was hard for them to find time for the intervention during a busy working day. However, a number of nurses also felt relieved that patients in the intervention group dispensed and self-administered their own medication.

Nurses generally adhered to the study protocol. However, at the time of admission, the nurses often forgot to assess new patients’ capability of self-administration. The PI tried to recruit these patients the following day, but many were already about to be discharged making the intervention impossible. Secondly, the nurses should deliver a whole package of medication to the patient if new medication was prescribed or the patient had not brought medication to the hospital; however, if the medication was available in blister packages, the nurses often delivered this instead of a whole package, despite information and instructions about the intervention. As with other complex interventions [17], continuous project management and training of new staff will be necessary during the RCT.

### Recruitment and acceptability

A total of 60 patients were recruited within 3 months as planned, and overall, the recruitment was considered satisfactory. However, 14% of the invited patients declined to participate for unknown reasons. The reasons for declining to participate must be registered in the RCT as it is important to know if the intervention is not accepted among those patients. As mentioned above, many patients were not assessed for eligibility before it was too late for inclusion. Eligibility information was captured by the PI in dialog with nurses and by reviewing electronic medical records. As patients are hospitalized for a short time, the assessment of eligibility must be prioritized to recruit more patients. Medication reconciliation is performed in, for example, the Emergency Department prior to the referral to the Cardiology Unit. The assessment of SAM capability could be performed in this stage; however, some acutely ill patients may be cognitive impaired. To reduce complexity of the RCT study setting, it was considered better to focus on only one unit.

Monday to Thursday, nurses had to recruit new patients when the PI was not present. Eleven out of 60 patients (18%) were recruited in weekends and holidays, making observation of dispensing impossible. For practical and ethical reasons, this number must be minimized in the RCT. One way of doing this is to make the PI or a research assistant responsible for the inclusion. This will be considered for the RCT.

Twenty-three of the patients were capable of self-administration but were excluded because they did not bring their own medication to hospital. It is considered that it may be easier for patients in elective admissions to bring in their own medication and pre-consent with advanced notice. On the other hand, some patients admitted acutely may also be capable of and benefit from SAM, even if they did not bring medication to the hospital. In that case, there may not be savings for the hospital on medication, but SAM may still provide savings on nursing time as well as patient benefits. We therefore decided to keep the patient group for the RCT fairly open to patients capable of SAM and switching to another study setting was not in consideration. However, to increase the number of eligible patients and ensure recruitment, the exclusion criterion “did not bring any of their medication” is considered to be removed in a future RCT.

A pragmatic RCT is considered to ensure recruitment and because the intervention has to be tested in a real-world clinical practice setting.

The intervention was highly accepted by patients, and most of them preferred to have the opportunity to administer their own medication in a possible future hospitalization. The questions regarding acceptability were not tested for face validity; this must be done before the RCT. A high score of patient satisfaction concerning self-administration of medication was also found in another Danish study among 66 gastric surgery and acute orthopedic surgery patients [25].

### Outcome measurement methods

The modified disguised observation technique detected errors in both groups. From previous research, it is well known that the observer may affect the person being observed (“the Hawthorne effect”); however, this bias has limited importance, since the observed person often get used to being observed and thus demonstrates usual behavior [1, 4, 28]. Procedural errors were not observed in the intervention group. Initially, it was considered irrelevant since it was expected that the nurses followed the intervention guideline which was developed by their own nurses. However, in order to be comparable, the same types of errors must be observed in both groups in the RCT, including both clinical and procedural errors. The clinical errors are the most important ones to avoid; however, since the study compares workflows and as procedural errors have the potential to cause harm [5, 26], it is considered relevant to measure procedural errors as well.

The observations of the dispensing process were repeated up to four times per patient because of a concern that the patients were not informed about prescription changes. The number of observations per patient must be more structured in a future RCT, as the groups must be comparable and data dependence must be minimized. Observation at least once and no more than twice is therefore considered relevant in a future RCT.

Previous research has shown mixed results of the impact of SAM on adherence [14]. It is considered relevant to measure effects after discharge as secondary outcomes, e.g., perception about medication [31], which correlates to medication adherence [32]. Patient involvement initiatives are expected to improve patients’ knowledge, skills, and confidence [7]. To gain evidence on SAM patient perspectives (e.g., their confidence), a qualitative study is planned as supplement of the RCT.

### Limitations

Beforehand, it was decided to include 60 patients; the first 40 in the intervention group and the last 20 patients were to be randomized at a 1:1 ratio. This design made it possible to gain considerable experience with the intervention in only 3 months. However, it also limited the number of patients and thereby the number of observations of the dispensing process in the control group. This may have influenced the error proportion (16%) used for calculating the required sample size in the RCT. Hence, it can be discussed whether it is adequate to base a sample size calculation on only nine patients. In comparison, a recent Danish study investigating the impact of an automatic medication system found an error proportion of 17% in the control group (nurse-led dispensing) [5]. An error proportion of 16% is therefore considered a realistic estimate for the sample size calculation.

The intervention group was not observed for procedural errors, as it initially was considered irrelevant; thus, the total error proportion cannot be compared between groups. In the RCT, both groups must be observed for the same types of errors.

It has been suggested that the obtained medication history at admission is more precise when the patients bring their own medication to hospital [33]. Thus, an advantage of bringing own medication to hospital may be a reduced number of medication reconciliation errors; however, this was outside the scope of this study and the following RCT.

We acknowledge the risk of selection bias by not including patients in weekends and holidays, as patients are often more ill when admitted during the weekends [34]. For practical reasons, patients were mainly included from Monday to Thursday. With inclusion only performed on working days, it will remain unclear whether SAM is suitable for patients admitted during weekends. Dispensing was primarily observed in the mornings; however, other medication routines may have existed during the day. Therefore, our results may not be comparable to medication routines at other times of the day.

Due to the intervention type, blinding to group allocation was not possible. This may have increased the risk of bias, e.g., in the collection of data, and thus give a risk of overestimating the effect.

## Conclusions

In conclusion, the present study shows that it may be feasible to perform a pragmatic RCT of the safety and cost-consequences of SAM. Only minor adjustments are considered for the RCT.

## Supplementary information

**Additional file 1:.** CONSORT pilot feasibility checklist.

**Additional file 2:.** Questions at follow-up.

## Data Availability

The datasets used and/or analyzed during the current study are available from the corresponding author on reasonable request.

## Abbreviations

CI: Confidence interval
eMAR: Electronic Medication Administration Record
MAEs: Medication administration errors
GP: General practitioner
OEs: Opportunities for error
PI: Primary investigator
RCT: Randomized controlled trial
SAM: Self-administration of medication
SD: Standard deviation
