# Supplementary material for: Self-administration of medication during hospitalization—a randomized pilot study
Source: Pilot Feasibility Stud. 2020 Aug 18;6:116. doi: 10.1186/s40814-020-00665-3 (PMC7433129; doi:10.1186/s40814-020-00665-3)
Supplement: Supplementary file 2 — Additional file 2:. Questions at follow-up. [file 40814_2020_665_MOESM2_ESM.pdf]

Additional file 1: Structured guide of questions at follow-up

**Question**	**Response categories**
Theme 1: Healthcare use since discharge from the Cardiology unit, Randers Regional Hospital	
Have you been admitted to a hospital since you were discharged from the Cardiology unit, Randers Regional Hospital?	Yes/No
If yes, do you remember when?	Date:
Have you visited your general practitioner since you were discharged from the Cardiology unit, Randers Regional Hospital?	Yes/no
If yes, do you remember when?	Date:
Theme II: Perception of medication management at the Cardiology unit, Randers Regional Hospital	
Please rate your level of satisfaction with the way you received medication during your hospitalization in the Cardiology unit, Randers Regional Hospital?	1 – very unsatisfactory<br>2 – unsatisfactory<br>3 – Neither/nor<br>4 – Satisfactory<br>5 – very satisfactory
Do you prefer self-administration of your medication in a possible future hospitalization?	Yes/No
